# Protein–macrocycle polymorphism: crystal form IV of the *Ralstonia solanacearum* lectin–sulfonato-calix[8]arene complex

**DOI:** 10.1107/S2059798323003832

**Published:** 2023-06-14

**Authors:** Niamh M. Mockler, Kiefer O. Ramberg, Peter B. Crowley

**Affiliations:** a School of Biological and Chemical Sciences, University of Galway, University Road, Galway H91 TK33, Ireland; University of Oxford, United Kingdom

**Keywords:** β-propellers, crystal engineering landscape, lectins, protein assembly, sulfonato-calix[8]arene form IV, *Ralstonia solanacearum*

## Abstract

Controlled protein assembly and crystallization is necessary as a means of generating diffraction-quality crystals as well as providing a basis for new types of biomaterials. Water-soluble calixarenes are useful mediators of protein crystallization. Recently, it was demonstrated that *Ralstonia solanacearum* lectin (RSL) co-crystallizes with anionic sulfonato-calix[8]arene (sclx_8_) in three space groups. Two of these co-crystals only grow at pH ≤ 4 where the protein is cationic, and the crystal packing is dominated by the calixarene. This paper describes a fourth RSL–sclx_8_ co-crystal, which was discovered while working with a cation-enriched mutant. Crystal form IV grows at high ionic strength in the pH range 5–6. While possessing some features in common with the previous forms, the new structure reveals alternative calixarene binding modes. The occurrence of *C*_2_-symmetric assemblies, with the calixarene at special positions, appears to be an important result for framework fabrication. Questions arise regarding crystal screening and exhaustive searching for polymorphs.

## Abstract 2

Calixarene-mediated protein assembly provides a basis for the development of new types of biomaterials. A fourth structure of the *Ralstonia solanacearum* lectin–sulfonato-calix[8]arene complex expands the crystal-engineering landscape and suggests an alternative pH trigger of assembly.

## 1. Introduction

In a recent editorial, Desiraju remarked on the conceptual shift from *the structure* to *a structure*, the latter being a point in the crystal-engineering landscape of a small molecule (Desiraju, 2021 ▸). Notwithstanding some discussion regarding definitions (Jones & Ulrich, 2010 ▸; Ulrich & Pietzsch, 2015 ▸), protein crystal polymorphism is a well recognized phenomenon (Ebrahim *et al.*, 2019 ▸; Gillespie *et al.*, 2014 ▸; Lanza *et al.*, 2019 ▸; Van Driessche *et al.*, 2018 ▸). The tetrameric d-glucose isomerase (∼180 kDa) crystallizes in space group *I*222 or *P*2_1_2_1_2 under low or high precipitant concentrations, respectively (Gillespie *et al.*, 2014 ▸; Van Driessche *et al.*, 2018 ▸; Vuolanto *et al.*, 2003 ▸). Despite differences in the crystallization mechanisms, either ammonium sulfate (salting-out) or polyethylene glycol (depletion attraction) can act as the precipitant. Chicken egg-white lysozyme (∼14 kDa), which is possibly the best crystallographically characterized protein, with ∼1000 entries in the Protein Data Bank (PDB), crystallizes in at least six space groups (most frequently in *P*4_3_2_1_2), with some evidence that anion binding can select the space group (Lanza *et al.*, 2019 ▸; Plaza-Garrido *et al.*, 2018 ▸; Vaney *et al.*, 2001 ▸; Zalar *et al.*, 2023 ▸). Co-crystals of the polyanionic sulfonato-calix[4]arene with lysozyme or methylated lysozyme reveal the polyanion to play key roles in the crystal packing (McGovern *et al.*, 2014 ▸, 2015 ▸).

**Figure 1:**
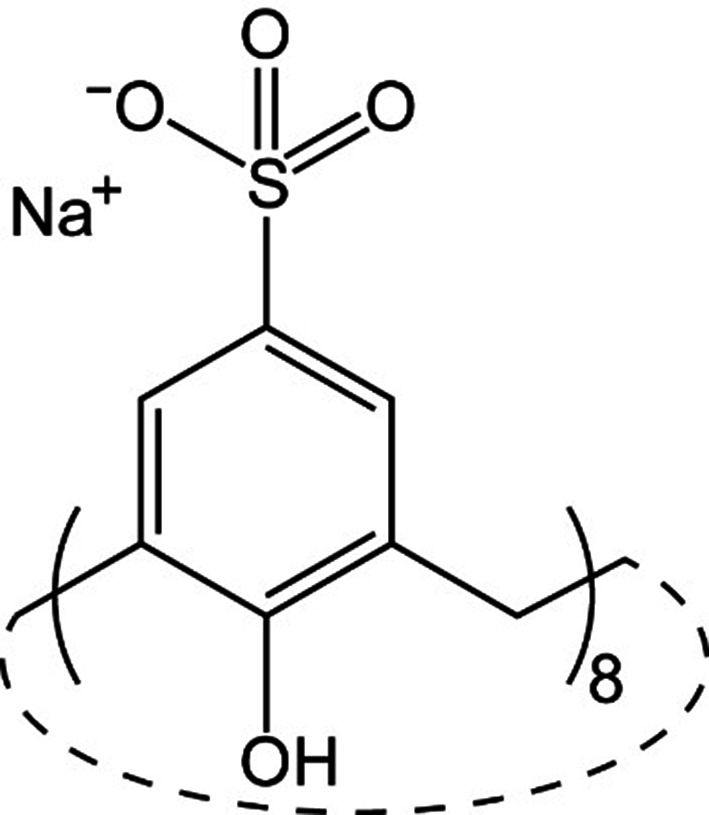
The sulfonato-calix[8]arene (sclx_8_) macrocycle with sodium counterions.

Calix[*n*]arenes are macrocyclic polyphenols with extensive crystal-engineering applications (Atwood *et al.*, 2002 ▸; Kravets *et al.*, 2021 ▸; Leśniewska *et al.*, 2019 ▸; Pasquale *et al.*, 2012 ▸). The sulfonato-calixarenes are versatile receptors for protein surfaces (with millimolar to micromolar binding affinities), acting as molecular glues with pronounced co-crystallization properties akin to ‘silver bullets’ (Alex *et al.*, 2019 ▸; McPherson & Cudney, 2006 ▸). As mediators of controlled assembly, calixarenes can contribute to the fabrication of protein-based materials (Engilberge *et al.*, 2019 ▸; Ramberg, Engilberge, Skorek *et al.*, 2021 ▸; Rennie *et al.*, 2018 ▸; Zhu *et al.*, 2021 ▸). The controlled formation of stable, porous protein assemblies may be an enabling technology in the development of biocatalysts (Nguyen *et al.*, 2021 ▸). Previously, we reported three co-crystal forms of sulfonato-calix[8]arene (sclx_8_, 1.5 kDa; Fig. 1 ▸) and cationic yeast cytochrome *c* (∼13 kDa) (Engilberge *et al.*, 2019 ▸; Rennie *et al.*, 2018 ▸). One of these co-crystal forms is highly porous with ∼85% solvent content and is mediated exclusively by the macrocycle. The crystal packing is devoid of protein–protein contacts. We have also reported three co-crystal forms of sclx_8_ and the bacterial lectin *Ralstonia solanacearum* lectin (RSL; ∼29 kDa) (Ramberg, Engilberge, Skorek *et al.*, 2021 ▸). Two of these co-crystals are highly porous and rely on protein–calixarene–protein interfaces. Seemingly, sclx_8_ mediates different protein frameworks (or polymorphs) and functions as a tool for supramolecular isomerism consistent with ‘the existence of more than one type of network superstructure for the same molecular building blocks’ (Moulton & Zaworotko, 2001 ▸).

RSL has a trimeric, six-bladed β-propeller structure with *C*_3_ symmetry, an isoelectric point (pI) close to neutral and high thermal stability (Kostlánová *et al.*, 2005 ▸). Table 1 ▸ lists the three previously described crystal forms of RSL and sclx_8_ (Ramberg, Engilberge, Skorek *et al.*, 2021 ▸). Forms I and II were obtained using a commercial crystallization screen. Form III was originally obtained in an NMR sample (pH 4, no precipitant) after overnight storage in the fridge. Form I, a densely packed crystal in space group *P*2_1_3, grows at high ammonium sulfate concentrations and over a wide pH range. The requirement for high salt and the absence of pH dependence suggests that the hydrophobic effect dominates the formation of protein–calixarene and protein–protein interfaces. Crystal forms II (space group *I*23) and III (space group *P*3) grow at pH ≤ 4 where RSL is cationic, and charge–charge interactions are expected to dominate. Both forms II and III are porous and mediated exclusively by sclx_8_, with no protein–protein interfaces, emphasizing the molecular-glue capacity of sclx_8_. Each of the three RSL–sclx_8_ co-crystal forms involve calixarene binding by the key residues Val13 and Lys34, albeit with differences in the calixarene conformation.

In our previous study, several mutants and chemical modifications of RSL were also tested (Ramberg, Engilberge, Skorek *et al.*, 2021 ▸). The mutant MK-RSL with an extended N-terminus containing the Met–Lys motif that binds cucurbit[6]uril (Ramberg, Engilberge, Guagnini *et al.*, 2021 ▸) was hypothesized to bind sclx_8_. Trials using the form III co-crystallization condition were unsuccessful. The present work picked up at this point and a broad co-crystallization screen of MK-RSL and sclx_8_ led to the discovery of form IV, which also occurs with native RSL. Interestingly, the calixarenes are at special positions on crystallographic twofold axes. Calixarene binding at Val13 and Lys34 reoccurs but in a substantially altered format. The role of protein charge and pH screening is discussed in the context of the protein–macrocycle crystallization landscape.

## 2. Materials and methods

### 2.1. Materials

Stock solutions of sclx_8_ (Tokyo Chemical Industry) were prepared in water and the pH was adjusted to 7.5. RSL and MK-RSL, produced in *Escherichia coli* BL21 cells, were purified and quantified as described previously (Ramberg, Engilberge, Guagnini *et al.*, 2021 ▸; Ramberg, Engilberge, Skorek *et al.*, 2021 ▸). Each protein was studied in the d-fructose-bound form.

### 2.2. Co-crystallization trials

Solutions of ∼1 m*M* RSL in water or MK-RSL in 20 m*M* potassium phosphate, 50 m*M* NaCl pH 6.0 were co-crystallized with sclx_8_ at 20°C. MK-RSL was tested with 4, 16 or 32 m*M* sclx_8_ via sitting-drop vapour-diffusion experiments in MRC plates. Drops were prepared with a commercial screen (JBScreen JCSG++ HTS, Jena Bioscience) using an Oryx8 robot (Douglas Instruments). Hanging-drop vapour diffusion in 24-well Greiner plates was used to test solutions comprising RSL or MK-RSL and 32 m*M* sclx_8_ in combination with 1–2 *M* sodium citrate at pH 4–6 or unbuffered. Crystallization drops were imaged using an Olympus SZX16 stereomicroscope and an Olympus DP25 digital camera.

### 2.3. X-ray data collection, processing and model building

Crystals were cryoprotected in the crystallization solution supplemented with 20–25%(*v*/*v*) glycerol and cryocooled in liquid nitrogen. Diffraction data were collected at 100 K on the PROXIMA-2A beamline at the SOLEIL synchrotron, Saint-Aubin, France using an EIGER X 9M detector. Data were processed using the *autoPROC* pipeline (Vonrhein *et al.*, 2011 ▸) with integration in *XDS* (Kabsch, 2010 ▸) and scaling and merging in *AIMLESS* (Evans & Murshudov, 2013 ▸) and *POINTLESS* (Evans, 2011 ▸). *AIMLESS* was used to cut the data to 1.18 Å resolution, with *I*/σ(*I*) = 1.80. Structures were solved via molecular replacement in *Phaser* (McCoy *et al.*, 2007 ▸) using the RSL monomer (PDB entry 2bt9) as a search model. The coordinates of sclx_8_ (PDB ID EVB) and d-fructose (PDB ID BDF) were added to each model in *Coot* (Emsley *et al.*, 2010 ▸). Model building in *Coot* and refinement in *phenix.refine* (Adams *et al.*, 2010 ▸) were performed iteratively until no further improvements in the *R*_free_ or electron density could be made. The structures were validated in *MolProbity* (Williams *et al.*, 2018 ▸) and deposited in the PDB with accession codes 8c9y and 8c9z. *PDBePISA* was used to determine protein–calixarene interface areas (Krissinel & Henrick, 2007 ▸). *MAP_CHANNELS* was used to calculate crystal pore diameters (Juers & Ruffin, 2014 ▸).

## 3. Results and discussion

### 3.1. Form IV crystallization conditions and structure determination

**Figure 2:**
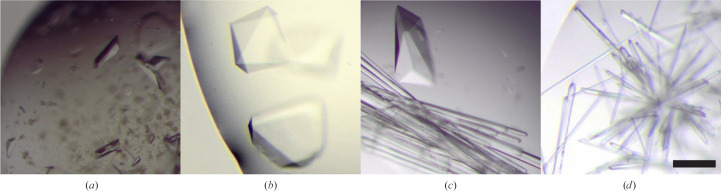
(*a*) MK-RSL–sclx_8_ co-crystals obtained in JBScreen JCSG++ HTS condition B11. (*b*, *c*, *d*) RSL–sclx_8_ co-crystallization trials in 1.0 *M* sodium citrate at pH 6, 5 or 4. Images are to scale and the scale bar is 100 µm in length.

The JBScreen JCSG++ HTS screen applied to mixtures of MK-RSL and sclx_8_ gave rise to crystals (Fig. 2 ▸ *a*) in condition B11, unbuffered 1.6 *M* sodium citrate (nominally pH ∼8), at 32 m*M* calixarene. Previous (Ramberg, Engilberge, Skorek *et al.*, 2021 ▸) and reiterated trials with RSL and sclx_8_ did not yield crystals in this condition. The pH of condition B11 *in situ* is unknown. In the case of MK-RSL, which is prepared in potassium phosphate buffer, it is likely that the crystallization condition is pH 6–7. In the case of RSL, which is prepared in water, the pH may be ∼7 or higher. The elevated pI of MK-RSL with respect to RSL further hinted that the protein net charge may be important for co-crystallization in this condition. Consequently, hanging-drop vapour-diffusion trials were prepared in 1–2 *M* sodium citrate buffered at pH 4–6. Rhombohedral crystals of dimensions of ∼150 µm appeared in 1–2 days at 1.0–1.2 *M* sodium citrate pH 6 or 5 (Fig. 2 ▸). This crystal morphology, while similar to that obtained with MK-RSL, was distinct from those reported previously for RSL and sclx_8_ (Ramberg, Engilberge, Skorek *et al.*, 2021 ▸). Two morphologies, including rod-shaped crystals, grew in drops at pH 5. At pH 4 only the rods were obtained. Similarly, at >1.4 *M* sodium citrate pH 5 or 6 only the rods grew.

**Figure 3:**
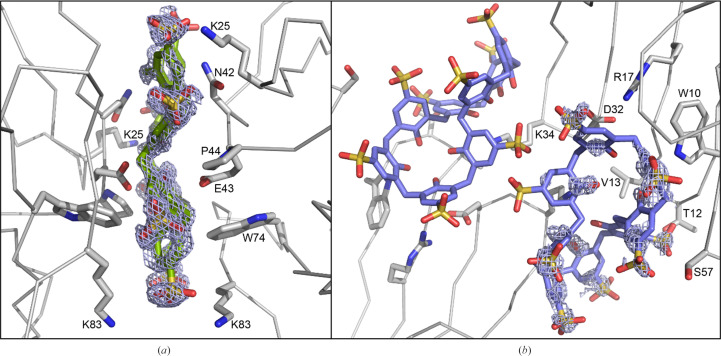
The RSL–sclx_8_ interfaces in crystal form IV showing the unbiased electron-density maps (2*F*_o_ − *F*_c_, calculated at 1.18 Å resolution prior to adding calixarenes to the model and contoured at 1σ). Two *C*_2_-symmetric interfaces are mediated by (*a*) one sclx_8_ molecule in the pleated loop and (*b*) two sclx_8_ molecules in a double-cone conformation. The sclx_8_ molecules are either wholly (*a*) or partly (*b*) located on a special position. Interface side chains are shown as sticks. For clarity, water molecules are omitted.

Diffraction data were collected at the SOLEIL synchrotron. The rod crystals proved to be sclx_8_ only. The crystals with MK-RSL or RSL diffracted to beyond 1.2 Å resolution and essentially identical structures were solved in space group *H*32 (Table 2 ▸) with electron density for sclx_8_ evident in the unbiased maps (Fig. 3 ▸). In the MK-RSL–sclx_8_ structure the extended N-terminus is disordered, with no electron density for either Met0 or Lys1. Thus, while this mutant aided the discovery of crystal form IV, the extended N-terminus apparently does not bind calixarene.

### 3.2. Calixarenes at special positions

Crystal form IV was solved in space group *H*32, with an asymmetric unit comprising one RSL monomer and two molecules of sclx_8_. Each calixarene is located at a special position on a crystallographic twofold axis. One sclx_8_ molecule occurs in the fully extended, pleated loop conformation, with all atoms on a special position and was modelled at 50% occupancy (Fig. 3 ▸ *a*). This sclx_8_ molecule is highly ordered with low average temperature factors (∼15 Å^2^) similar to those of the protein (∼18 Å^2^). The other sclx_8_ molecule, modelled at 70% occupancy, adopts a double-cone conformation (Fig. 3 ▸ *b*). This calixarene is less well defined (∼24 Å^2^), with three partly disordered phenol-sulfonate subunits, one of which is located on a special position.

We note two examples of protein–macrocycle co-crystal structures with features at special positions relevant to this study. A structure of concanavalin A in complex with tetrasulfonato-phenyl porphyrin (PDB entry 1jn2) solved in space group *F*222 includes half the porphyrin on crystallographic twofold axes (Goel *et al.*, 2001 ▸). A structure of *Rhodobacter capsulatus* bacterioferritin (PDB entry 1jgc) solved in space group *I*422 includes pseudo-*C*_2_-symmetric heme groups modelled at 50% occupancy on crystallographic twofold axes (Cobessi *et al.*, 2002 ▸).

### 3.3. Details of the calixarene binding sites

The pleated-loop sclx_8_ is nestled between two RSL trimers related by a 180° rotation (Fig. 3 ▸ *a*). Each protein buries ∼350 Å^2^ in the protein–sclx_8_–protein interface. Lys25, Asn42, Pro44 and Lys83 each contribute ≥45 Å^2^ to the interface area. The core of the interface is polar, involving Asn42, Glu43, the phenolic rim of sclx_8_ and several water molecules. Glu43 is likely to be protonated as it has an unusually high p*K*_a_ value due to coplanar stacking of the carboxyl group with the indole of Trp74 (Ramberg, Engilberge, Skorek *et al.*, 2021 ▸). A central water molecule, at a special position, is within van der Waals distance of all eight phenol hydroxyls and is hydrogen-bonded to the carbonyl backbone of Asn42 and the side chain of Glu43. Lys25 and Lys83, the side-chain termini of which are disordered, occur on the binding-site periphery, making weak salt-bridge interactions with the sulfonic acids.

The double-cone sclx_8_, although partly disordered, also interacts with two RSL trimers related by a 180° rotation. This assembly resembles the sclx_8_-mediated crystallographic dimer of *Penicillium* antifungal protein (PDB entry 6haj; Alex *et al.*, 2019 ▸), as well as features in sclx_8_–cytochrome *c* complexes (for example PDB entry 6rsi; Engilberge *et al.*, 2019 ▸; Rennie *et al.*, 2018 ▸). To describe the calixarene binding mode at this site we must reconsider the previously reported RSL–sclx_8_ structures (Ramberg, Engilberge, Skorek *et al.*, 2021 ▸). The β-propeller fold of the RSL monomer comprises two four-stranded antiparallel β-sheets. Val13 and Lys34 are located in adjacent loops of one of the sheets. In crystal forms I, II and III, Val13 and Lys34 are extensively encapsulated by sclx_8_ cavities comprising either two or three phenol-sulfonate subunits. The calixarene conformation at this site in crystal form IV most resembles that in form I, with four contiguous subunits superposing with an r.m.s.d. of <1 Å. However, in crystal form IV the double-cone sclx_8_ spans two RSL molecules binding Val13 in one trimer and Lys34 in the other trimer. The larger interface buries ∼350 Å^2^ of the protein, with major contributions (≥45 Å^2^) from Val13 and Ser57, while the smaller interface buries ∼180 Å^2^ of the second protein with Lys34 and Tyr37 as the main contributors. In this novel arrangement, Val13 forms CH–π bonds with just one phenol-sulfonate, and Lys34, while partly disordered, is within the vicinity of four phenolic hydroxyls. Overall, a *C*_2_-symmetric assembly is mediated by two adjacent sclx_8_ molecules and the junction of the two calixarenes (on a special position) is disordered. The model is approximate at the special position, with the phenol-sulfonates of the two molecules being interchangeable (Fig. 3 ▸ *b*). A curious consequence of the *C*_2_ symmetry and the 70% occupancy at this site comprising two calixarene bridging ligands is that the framework is maintained even if only one calixarene is present. Apparently, the molecular-glue capacity of one calixarene is sufficient to maintain this junction.

### 3.4. A comparison of RSL–sclx_8_ co-crystal frameworks

Table 1 ▸ shows the breadth of conditions leading to RSL–sclx_8_ co-crystals, all of which were obtained at ∼1 m*M* protein. Crystallization of forms II and III requires ≤10 equivalents of calixarene to protein. In contrast, forms I and IV require >30 equivalents. Such high concentrations of the octa-anionic calixarene greatly increase the ionic strength (30 m*M* Na^+^sclx_8_, is approximately 1.1 *M* ionic strength) and are likely to combine with the effects of ∼1 *M* precipitant (ammonium sulfate or sodium citrate) to achieve supersaturation. High ionic strength and a broad pH range leads to the densely packed form I (*P*2_1_3). In contrast, the porous forms II (*I*23) and III (*P*3) grow at pH 4 or lower, where RSL is cationic. The former grows at ∼3 *M* ionic strength while the latter requires low salt. Previously, we noted that a pH trigger involving the protonation of one or two Asp side chains enables forms II and III (Ramberg, Engilberge, Skorek *et al.*, 2021 ▸). Interestingly, crystal form IV appears to be a hybrid structure requiring a relatively narrow pH range (5–6) and high ionic strength. A pH trigger may also be relevant here. Glu43 is centrally located on either side of the calixarene glue, forming hydrogen bonds to two of the phenolic hydroxyls and to the central water molecule (Fig. 3 ▸ *a*). The two symmetry-related Glu43 side-chain carboxylates are separated by <5.5 Å. With a p*K*_a_ of ∼6 (Ramberg, Engilberge, Skorek *et al.*, 2021 ▸) this side chain is likely to be protonated, thus facilitating assembly of the protein–calixarene–protein junction. This proposed mechanism differs from the previously described pH trigger, in which calixarene binding was coupled to protonation of Asp32 and/or Asp46 at a pH of ∼4. Attempts to obtain crystal form IV at pH 4 failed. It is plausible that sclx_8_ consumption within the rod crystals (Fig. 2 ▸ *d*) compromised the growth of RSL–sclx_8_ co-crystals. Considering the high ionic strength, form IV is relatively porous, contrasting with the densely packed form I that also grew at high ionic strength. Porous cytochrome *c*–sclx_8_ frameworks were obtained at high ionic strength, for example >1.8 *M* ammonium sulfate (Engilberge *et al.*, 2019 ▸; Rennie *et al.*, 2018 ▸).

**Figure 4:**
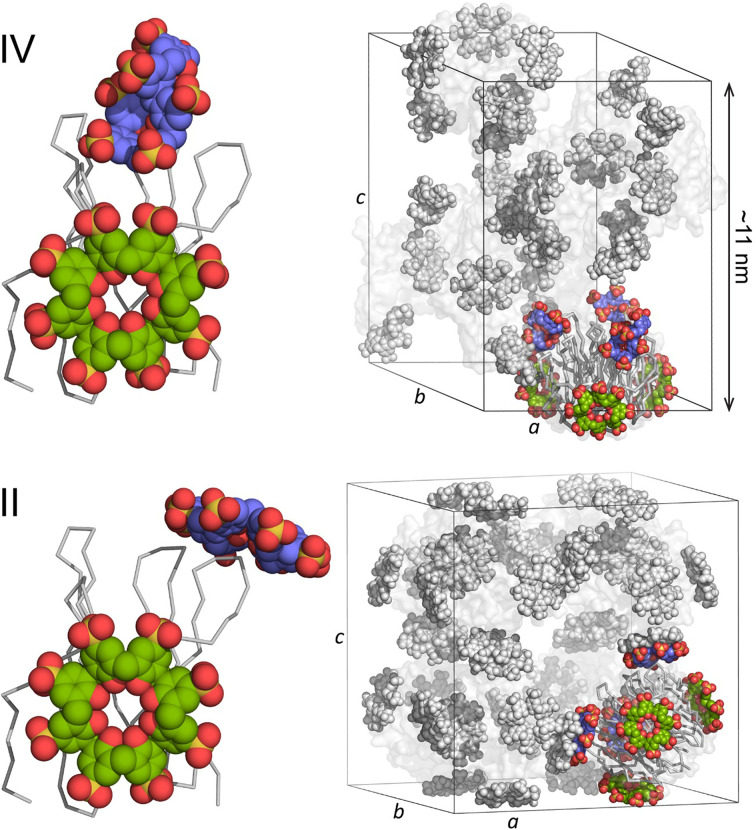
The asymmetric units and unit cells of RSL–sclx_8_ forms IV (*H*32) and II (*I*23). The unit cell is depicted with one RSL trimer in ribbon representation and the corresponding calixarenes in colour. The remaining components are in grey with proteins as transparent surfaces. The unit cells are drawn to scale and the approximate *c* dimension is indicated in form IV.

Forms III and IV have similar (trigonal) packing, with each protein trimer connected to six other trimers via calixarene junctions. The RSL trimer is a toroid (Kostlánová *et al.*, 2005 ▸), like a tube cake, with a wide end (∼4.5 nm) and a narrow end (∼2.5 nm). Lys34 is located at the wide end, Lys25 at the narrow end and Lys83 is midway between the two. In form III (*P*3) the wide ends and narrow ends are bridged together by calixarenes. Each protein trimer shares six calixarenes with symmetry-related proteins in the crystal packing. In form IV (*H*32), two symmetry-related calixarenes (Figs. 3 ▸ *b* and 4 ▸) mediate packing between the wide ends. Protein–calixarene–protein packing also occurs via the mid-regions of the toroids. Notwithstanding the reduced occupancy, each trimer effectively shares nine calixarenes with symmetry mates. Form I also involves nine shared calixarenes, albeit with several small (<90 Å^2^) interfaces. Form II (*I*23) has eight calixarene-coated trimers making up the cubic unit cell. In this packing, each RSL trimer shares 12 calixarenes (arranged as dimers) with symmetry mates. Thus, it appears that form IV is intermediate to forms II and III. Strikingly, form IV utilizes a new calixarene binding arrangement at the wide end of RSL, while the calixarene binding site at Lys25/Lys83 replicates a feature found in form II (Figs. 4 ▸ and 5 ▸). Fig. 4 ▸ shows the asymmetric units and unit cells of forms II and IV. The calixarenes coloured green are similar in the two structures. The calixarenes coloured mauve, although in different conformations, bind to similar regions of the protein. In form II, the two calixarenes dimerize to mediate the cubic packing (Figs. 4 ▸ and 5 ▸). In form IV, each calixarene acts as an independent molecular glue to mediate two distinct *C*_2_-symmetric interfaces. Apparently, the polymorph selection is controlled by the choice of precipitant (ammonium sulfate or sodium citrate), the ionic strength and the pH (Table 1 ▸).

## 4. Conclusions

The commercially available sclx_8_ is a versatile mediator of protein crystallization (Alex *et al.*, 2019 ▸; Engilberge *et al.*, 2019 ▸; Rennie *et al.*, 2018 ▸). This flexible macrocyclic anion can bind to the same protein surface in different ways, leading to distinct assemblies. Using RSL and sclx_8_ building blocks, four crystalline frameworks with a range of porosities (36–66% solvent content) can be generated (Table 1 ▸). Apparently, crystal engineering is relatively straightforward with selection via the choice/concentration of precipitant and the pH. Three of the co-crystal forms were discovered previously (Ramberg, Engilberge, Skorek *et al.*, 2021 ▸). Two of these were hits in a commercial screen, while the third occurred in an NMR sample. Crystal form IV was not obtained in the original trials with RSL because the effect of pH on the sodium citrate condition was not studied. Testing the cation-enriched variant MK-RSL led to the discovery of form IV. Future protein–calixarene co-crystallization trials will include focused testing in sodium citrate at pH 4–6. Such simple crystallization conditions are attractive in the context of protein-based materials and industrial applications.

While the strict definition of a polymorph, an ‘identical chemical composition but different crystal structure’, does not necessarily apply to protein crystals (Jones & Ulrich, 2010 ▸; Ulrich & Pietzsch, 2015 ▸), it is reasonable to assert that the RSL–sclx_8_ co-crystals are polymorphs. Crystal form IV is another point on the RSL–sclx_8_ crystal-engineering landscape. Form IV appears to be a hybrid with properties (including crystallization conditions, calixarene binding sites and crystal packing) intermediate to the original forms. It remains to be seen whether yet other polymorphs will be discovered. Interestingly, three of the four RSL–sclx_8_ co-crystal forms are porous (solvent contents ranging from 51% to 66%) and are mediated exclusively by the calixarene (Fig. 4 ▸). Such engineered frameworks hold promise for the design and development of new protein-based materials.

## Supplementary Material

PDB reference: MK-RSL–sulfonato-calix[8]arene complex, *H*32 form, 8c9y

PDB reference: RSL–sulfonato-calix[8]arene complex, *H*32 form, 8c9z

## Figures and Tables

**Figure 5:**
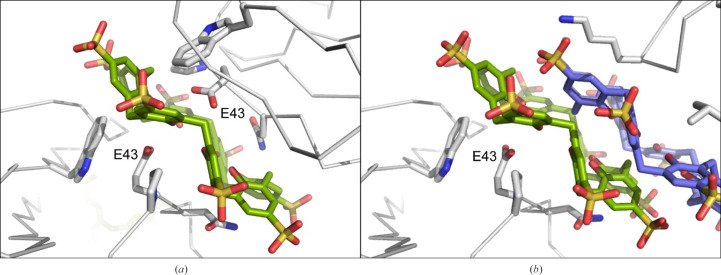
The RSL–sclx_8_ interfaces at Glu43 in crystal forms (*a*) IV (*H*32) and (*b*) II (*I*23). Glu43 and the flanking side chains Asn42, Pro44 and Trp74 are shown as sticks. For clarity, water molecules are omitted.

**Table 1:** RSL–sclx_8_ co-crystal forms and crystallization conditions

Form	[Salt]† (*M*)	*I* ‡ (*M*)	pH	Space group	PDB code§	*a*, *b*, *c* (Å)	sclx_8_ share¶	SC†† (%)	Pore diameter‡‡ (nm)
I	≥1.6	≥4.8	4.8–9.5	*P*2_1_3	6z60	64, 64, 64	9	36	1.7
II	0.8–1.0	2.4–3.0	≤4.0	*I*23	6z5g	104, 104, 104	12	66	4.2
III	None	∼0.1	≤4.2	*P*3	6z5q	60, 60, 64	6	59	2.8
IV	1.0–1.2	>4	5.0–6.0	*H*32	8c9z	76, 76, 114	9	51	2.7

† Approximate concentration of ammonium sulfate (forms I and II) or sodium citrate (form IV) at 1 m*M* protein.

‡ Approximate ionic strength of reservoir components.

§ Representative PDB entries.

¶ The number of sclx_8_ molecules per RSL trimer in the crystal packing.

†† Solvent content estimated from total mass (protein plus sclx_8_).

‡‡ Diameter of the widest pore calculated in *MAP_CHANNELS*.

**Table 2:** Crystallization conditions and X-ray data-collection, processing and refinement statistics for crystal form IV of RSL–sclx_8_ and MK-RSL–sclx_8_

Structure	RSL–sclx_8_	MK-RSL–sclx_8_
Sequence	SSVQTAATSWGTVPSIRVYTANNGKITERCWDGKGWYTGAFNEPGDNVSVTSWLVGSAIHIRVYASTGTTTTEWCWDGNGWTKGAYTATN	**MK**SVQTAATSWGTVPSIRVYTANNGKITERCWDGKGWYTGAFNEPGDNVSVTSWLVGSAIHIRVYASTGTTTTEWCWDGNGWTKGAYTATN
Crystallization		
[Sodium citrate] (*M*)	1.2	1.6
pH	6.0	Unknown
Data collection		
Light source	PROXIMA-2A, SOLEIL	PROXIMA-2A, SOLEIL
Wavelength (Å)	0.98011	0.98011
Temperature (K)	100.0	100.0
Space group	*H*32	*H*32
*a*, *b*, *c* (Å)	76.114, 76.114, 113.659	75.933, 75.933, 113.851
α, β, γ (°)	90.0, 90.0, 120.0	90.0, 90.0, 120.0
Resolution (Å)	57.02–1.18 (1.20–1.18)	56.94–1.18 (1.20–1.18)
No. of reflections	722097 (17558)	633389 (15353)
No. of unique reflections	41706 (1963)	41673 (2023)
Multiplicity	17.3 (8.9)	15.2 (7.6)
〈*I*/σ(*I*)〉	19.0 (1.8)	20.4 (1.8)
Completeness (%)	99.8 (96.3)	100.0 (99.8)
*R*_meas_ (%)	6.7 (138.3)	5.8 (131.4)
*R*_p.i.m._ (%)	1.6 (44.8)	1.5 (46.4)
CC_1/2_	1.000 (0.622)	1.000 (0.697)
Solvent content (%)	51	51
Refinement		
*R*_work_	0.165	0.163
*R*_free_	0.178	0.175
R.m.s.d., bond lengths (Å)	0.005	0.005
R.m.s.d., angles (°)	0.852	0.769
No. of molecules in asymmetric unit		
Protein chains	1	1
sclx_8_	2	2
Waters	103	93
Average *B* factor (Å^2^)	18.93	20.09
Clashscore	3.59	2.96
Ramachandran analysis, residues in		
Favoured regions (%)	98.86	98.88
Allowed regions (%)	1.14	1.12
PDB code	8c9z	8c9y
